# Unraveling the roles of genotype and environment in the expression of plant defense phenotypes

**DOI:** 10.1002/ece3.7639

**Published:** 2021-06-08

**Authors:** Abigail S. Potts, Mark D. Hunter

**Affiliations:** ^1^ Department of Ecology & Evolutionary Biology University of Michigan Ann Arbor MI USA

**Keywords:** cardenolides, defoliation, genetic variation, genotype‐by‐environment interactions, latex, milkweed, plant defense, resistance traits

## Abstract

1. Phenotypic variability results from interactions between genotype and environment and is a major driver of ecological and evolutionary interactions. Measuring the relative contributions of genetic variation, the environment, and their interaction to phenotypic variation remains a fundamental goal of evolutionary ecology.
2. In this study, we assess the question: How do genetic variation and local environmental conditions interact to influence phenotype within a single population? We explored this question using seed from a single population of common milkweed, *Asclepias syriaca*, in northern Michigan. We first measured resistance and resistance traits of 14 maternal lines in two common garden experiments (field and greenhouse) to detect genetic variation within the population. We carried out a reciprocal transplant experiment with three of these maternal lines to assess effects of local environment on phenotype. Finally, we compared the phenotypic traits measured in our experiments with the phenotypic traits of the naturally growing maternal genets to be able to compare relative effect of genetic and environmental variation on naturally occurring phenotypic variation. We measured defoliation levels, arthropod abundances, foliar cardenolide concentrations, foliar latex exudation, foliar carbon and nitrogen concentrations, and plant growth.
3. We found a striking lack of correlation in trait expression of the maternal lines between the common gardens, or between the common gardens and the naturally growing maternal genets, suggesting that environment plays a larger role in phenotypic trait variation of this population. We found evidence of significant genotype‐by‐environment interactions for all traits except foliar concentrations of nitrogen and cardenolide. Milkweed resistance to chewing herbivores was associated more strongly with the growing environment. We observed no variation in foliar cardenolide concentrations among maternal lines but did observe variation among maternal lines in foliar latex exudation.
4. Overall, our data reveal powerful genotype‐by‐environment interactions on the expression of most resistance traits in milkweed.

## Graphical abstract

This study explores how local growing environment and genetic variation dictates plant trait expression within a small population. We measured within‐population genetic variation in resistance traits of common milkweed and then compared those traits with patterns of resistance in the naturally growing milkweed population. Our findings suggest that genotype‐by‐environment interactions and environmental variation strongly influence phenotypic expression in this population of common milkweed and that standing genetic variation alone has a limited role in phenotypic variation.

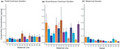

## 1 INTRODUCTION

To understand the significance of an organism's phenotype, and consequently its interactions with organisms around it, we must understand the independent and interactive effects of both the organism's genotype and its environment in determining that phenotype. Phenotypic variability results from both genotype and environment and is an important driver of many ecological and evolutionary processes (Hahn et al., 2019; Via & Lande, 1985; Zirbel & Brudvig, 2020). Genetic variation is well known to have substantial effects on trait expression (Agrawal & Hastings, 2019a; Geber & Griffen, 2003; Mousseau & Roff, 1987), providing the variation on which selection may act during evolution (Baucom & Mauricio, 2004; Burger & Lynch, 1995). Environmental variation, in addition to its role in contributing to phenotypic variation (Couture et al., 2015; Davis et al., 2005; Decker et al., 2019), is a source of strong selection on certain phenotypes, thus influencing which genotypes may prosper (Beemelmanns & Roth, 2017; Jay et al., 2012; Vannette & Hunter, 2011). When environments change, populations may become mal‐adapted (Ibáñez et al., 2010; Jay et al., 2012; Patankar et al., 2013; Sorte et al., 2013), and, if the population lacks genetic variation, the threat of extinction looms (Burger & Lynch, 1995).

Plant–herbivore interactions provide interesting systems for investigating the causes and consequences of phenotypic variation because they are ubiquitous in terrestrial systems and mediate numerous indirect effects with other herbivores (Ali & Agrawal, 2014), pollinators (Moreira et al., 2019), soil microbial associates (Peschel et al., 2015), and other trophic levels (Hunter, 2016; Price et al., 1980). A substantial amount of phenotypic variation exists within and among plant species, and this variation influences species interactions (Agrawal & Hastings, 2019b; Bucharova et al., 2017; Coley, 1987; Wetzel et al., 2016, 2018). Because plants are sessile during significant portions of their lifecycles, their populations experience strong selection to adapt to the local environment (Bossdorf et al., 2005; Bucharova et al., 2017; Cipollini, 2002; Jay et al., 2012; Weißhuhn et al., 2012), including in defense traits against their herbivores (Agrawal, 2005; Agrawal & Van Zandt, 2003; Coley, 1987; Vannette & Hunter, 2011).

The degree to which plant defenses are heritable varies considerably (Agrawal et al., 2002; Wooley et al., 2007), and plant defenses also vary with environmental conditions (Decker et al., 2018; Hahn & Maron, 2018; Mondor et al., 2006; Ode et al., 2014), including insect attack (Ali & Agrawal, 2014; Howe & Schaller, 2008). However, plant responses to environmental conditions are dependent on genetic variation (Des Marais et al., 2013; Lehndal & Ågren, 2015). The interplay between genotype and environment (genotype‐by‐environment interactions; G × E) is well known to drive ecological interactions (Des Marais et al., 2013; Fritz & Price, 1988; Saltz et al., 2018; Vannette & Hunter, 2011). However, it is often difficult to determine the degree to which genetics and the environment are responsible for particular aspects of phenotypic variation (Maddox & Root, 1987; Muola et al., 2010).

Evolutionary and ecological variation in plant defenses against herbivory are therefore countered in part by herbivore choices and adaptations (Birnbaum & Abbot, 2018). Mobile herbivores are able to make choices about their location and food source (Jones & Agrawal, 2019; Murphy & Loewy, 2015): An herbivore can lower consumption in response to increased toxicity (Whitehead & Poveda, 2011; Whitehead et al., 2016), continue to consume the plant but face consequences of decreased health and fitness (Tao, Hoang, et al., 2016), or even sequester the toxin for its own purposes (Jones et al., 2019; Petschenka & Agrawal, 2015). If environmental conditions change, thereby changing plant defense traits, we might expect herbivory patterns to change as well (Ode et al., 2014).

Determining the proportion of phenotypic variation that is attributable to genetic factors helps to predict the ability of populations to adapt to a rapidly changing environment (Via & Lande, 1985). Anthropogenic environmental change is reducing global insect populations (Brower et al., 2012; Hallmann et al., 2017), reducing native plant populations (Scheper et al., 2014), and changing plant phenotypes (Tao et al., 2014; Vannette & Hunter, 2011). When environmental conditions change rapidly, population adaptation depends on natural selection to act on heritable genetic variation (Via & Lande, 1985). In this study, we assess how genetic variation and local environmental variation combine as drivers of phenotypic variation and plant–herbivore interactions in a naturally growing population of *Asclepias syriaca*, common milkweed.

## 2 SYSTEM OF STUDY

Milkweeds (Apocynaceae) and their herbivores have become a model system for studying the ecology and evolution of plant–herbivore interactions (Brower et al., 1968; Hahn et al., 2019; Malcolm, 1994; Meier & Hunter, 2019; Zehnder & Hunter, 2007). Milkweed grows clonally in genetic individuals (genets) (Woodson, 1954) and these genets resist herbivory through several defensive traits: cardenolides, a group of cardiac glycoside steroids (Malcolm, 1991); latex, a sticky substance that inhibits chewing herbivores (Zalucki & Malcolm, 1999); and trichomes, hair‐like structures that impede herbivore feeding (Agrawal, 2004a; Levin, 1973). A group of specialist herbivores, including the monarch butterfly, *Danaus plexippus,* and several aphid species, have evolved to tolerate milkweeds' defenses (Agrawal, 2012; Ali & Agrawal, 2014; Birnbaum & Abbot, 2018; Sternberg et al., 2012).

Milkweeds display both inter‐ and intraspecific variation in defensive phenotypes (Agrawal & Hastings, 2019b; Hahn et al., 2019; Zehnder & Hunter, 2007), which in turn influence their ecological interactions (Birnbaum & Abbot, 2018; Zalucki et al., 1990). Variation in milkweed defense phenotypes is also driven by environmental variation (Decker et al., 2018; Meier & Hunter, 2019; Ricono et al., 2020; Tan et al., 2018; Tao, Ahmad, et al., 2016; Tao & Hunter, 2013). Global environmental change has greatly impacted the ecology of milkweeds (Malcolm, 2017) and their specialized herbivores (Decker et al., 2018; Pleasants & Oberhauser, 2013).

In this study, we ask the question: How do genetic variation and local environmental conditions interact to influence phenotype within a single population? To explore this, we grew *A*. *syriaca* in three experiments: a field common garden, a greenhouse common garden, and a reciprocal field transplant. Common gardens create a single environment in which plants are grown, and therefore, any differences among groups are inferred to reflect genetic variation (Agrawal & Van Zandt, 2003; Cipollini, 2002; Pellissier et al., 2016). Reciprocal transplants complement common garden experiments by allowing comparison of phenotypes of several genotypes across multiple environments, revealing effects of local environment on trait expression (Bucharova et al., 2017; Geber & Griffen, 2003). We then compared the data from the common gardens with data from the local population of *A. syriaca* from which the common garden seeds originated (referred to as “maternal genets” hereafter) to compare relative effects of genetic and environmental variation (Fritz & Price, 1988). By employing these three experiments and comparing the results to those from the unmanipulated maternal genets, this study assesses the contributions of genotype and environment to the expression of resistance phenotypes within a single milkweed population.

## 3 METHODS

### 3.1 Overall experimental design

To explore how milkweed genotype and environment influence resistance phenotypes, we designed three experimental groups: a field common garden, a greenhouse common garden, and a reciprocal field transplant. The seeds for all experiments came from fourteen genets of *A. syriaca*, growing in a 5‐acre old‐field at the University of Michigan Biological Station (UMBS) in Pellston, MI (45.558605, −84.677488). Within the old‐field, the growing environments of the genets vary in plant community cover type, proximity to structures and dirt roads, and proximity to the forest edge (M.D. Hunter, personal observations). The old‐field is maintained by semi‐annual mowing and hosts 32 genets that have been studied annually since 2007 (M.D. Hunter, personal observations). The 14 genets that we selected for our experiments were those that produced enough seed in 2018 for all three experiments in 2019. The distance between neighboring genets varies from approximately 5 m–20 m. The seeds were at least half‐siblings (multiple seed pods from unknown fathers for each genetic mother). Seeds and seedlings were classified by their maternal genotype and are referred to as “maternal lines” hereafter.

The field common garden and greenhouse common garden were both randomized block designs, and the same experimental design was replicated in the field and greenhouse. The reciprocal field transplant consisted of three maternal lines, each grown “at home” and “away.”

We measured defoliation, arthropod abundances, and plant size (height, leaf number, stem diameter) weekly from all common garden plants (12 weeks from 3 June–21 August 2019) and reciprocal transplant plants (10 weeks from 19 June–21 August 2019) and monthly from the naturally growing maternal genets. We collected samples for foliar chemistry from all plants once in mid‐July, in the middle of the growing season (methods below). This set of traits represents known drivers of insect performance, but we acknowledge that many other plant traits we did not sample likely also contribute to the insect abundance recorded in this study.

### 3.2 Common gardens

#### 3.2.1 Growing the plants

Plants for the field and greenhouse common gardens were grown from seed for one month at the University of Michigan in Ann Arbor before transfer to UMBS. Seeds were cold‐stratified for 6 weeks, treated with household bleach (5%), germinated in petri dishes for 1 week, and then planted in Sungro Metro‐mix^®^ 360 potting soil in Deepots^®^. Seedlings were grown in a controlled growth room (14:10 L:D, mean temperature 78°F) for the month of April 2019. Seeds were planted in April to ensure that plants were large enough to withstand field conditions by June, when local ramets emerge at UMBS. We transported plants from Ann Arbor to UMBS on 1 May 2019 to complete an additional month of greenhouse growth while outside conditions were still too cold. Plants were then either maintained in the greenhouse (greenhouse common garden) or transferred outside to the field common garden on 1 June 2019. This timing matches the typical phenology of the local milkweed population at UMBS (M.D. Hunter, *personal observations*).

#### 3.2.2 Experimental setup

The randomized block design of 18 blocks, each containing one individual of 14 maternal lines resulted in 252 plants total per common garden. Each plant was grown in an 18 cm × 16 cm pot held on benches (greenhouse) or set into the ground such that the topsoil of the pot was level with the ground (field). Each block consisted of two rows of 7 plants. Within each block of the field common garden, plants were spaced 1 m apart and 1.5 m separated each block. A 12.68 m × 32.53 m fenced exclosure surrounded the field common garden to protect plants from deer and rabbit browsing. The greenhouse common garden plants were arranged on benches so that plants were not touching. Plants were watered ad libitum and fertilized using Osmocote controlled‐release fertilizer (14:14:14 N:P:K) (ICL Specialty Fertilizers, Dublin, OH) once in May and once in July. From each *A. syriaca* plant, we measured weekly arthropod abundance and plant height, leaf number, defoliation, and base stem diameter (12 weeks total). In mid‐July, we measured foliar latex exudation and collected tissue to measure foliar concentrations of cardenolides, carbon, and nitrogen.

### 3.3 Reciprocal transplant experiment

We chose three of the 14 maternal genets to provide seed for reciprocal field transplants. We chose genets that spanned the known range of nutritional quality in *A. syriaca* at UMBS and also originated from spatially separated locations (at least 50 m apart) within the UMBS population. Based on the past 10 years of sampling, genet 14 has relatively high foliar nitrogen concentrations (3.60% N, 42.96% C), genet 20 has relatively low foliar nitrogen concentrations (2.72% N, 43.00% C), and genet 44 has relatively high foliar carbon concentrations (2.99% N, 44.34% C) (M.D. Hunter, *unpublished data*). Seeds from each of the three maternal lines were planted in the soil and location of all three of the original maternal genets. We chose to use only 3 maternal lines and 5 replicates at each location to minimize disturbance on the milkweed population growing in the old‐field. At each of the three maternal genet growing locations, 5 seedlings from each of the three maternal lines (15 seedlings total) were grown in soil from that maternal location (i.e., maternal location includes the maternal soil). Therefore, at each maternal location, we grew offspring plants from the “matching” maternal line (“at home” seedlings) and two “non‐matching” maternal lines (“away” seedlings).

Because we wanted to use soil from each maternal genet location, the reciprocal transplant experiment started later than the common garden experiments. Seeds were planted in 18 cm × 16 cm pots on 13 May 2019 at UMBS in the soil of their reciprocal transplant destination. Seedlings were grown in the greenhouse until large enough to withstand outside conditions and were placed in the field on 19 June. Replicate seeds per maternal line were established within the spatial boundaries of each of the maternal genets. Each transplant location (maternal genet location) hosted 5 replicate plants of each of 3 maternal lines, totaling 15 plants at each maternal genet location (45 plants total in the experiment) in a 3 × 5 plant grid with 0.5 m separating each plant. We randomized the order of the plants at each of the three locations. Plants were protected by a wire open‐top cage to block deer and rabbit browsing but allow access by insects. We measured arthropod abundance and plant height, leaf number, and defoliation weekly (10 weeks from 19 June to 21 August 2019) for each *A. syriaca* plant; foliar chemistry samples were collected once on 21 August. Stem diameter was not measured due to the small size of plants.

### 3.4 Maternal genet sampling

To measure trait variation in the naturally growing milkweed population, we sampled 5 individual ramets (randomly selected in June) from each of the 14 maternal genets (70 ramets total) on three dates (mid‐June, mid‐July, and mid‐August, 2019). We measured size (height, leaf number, stem diameter), defoliation, and arthropod abundance for each ramet. We collected foliar chemistry samples and measured latex exudation once in mid‐July.

### 3.5 Estimate of defoliation

To assess the contributions of genetic variation and environment to resistance to herbivory, we estimated defoliation by chewing herbivores from each plant in the common gardens, the reciprocal transplant experiment, and the maternal genets. We visually categorized each leaf longer than 1 cm into one of the following defoliation levels: no defoliation, 0%–5%, 5%–30%, 30%–50%, 50%–70%, 70%–90%, >90% defoliated. To estimate the overall percentage of defoliation per plant, we multiplied the number of leaves in each defoliation level by the median value of the level (2.5, 17, 40, 60, 80, 95) and summed the values. This sum was then divided by the total number of leaves on that plant. The final value represents the overall estimation of percent defoliation for that plant. This method has a long history in the literature and correlates strongly with independent estimates of defoliator activity (Hunter, 1987; Hunter et al., 1997; Meier & Hunter, 2019).

### 3.6 Plant chemical analyses

We performed chemical analyses (cardenolides, C:N) on foliar samples from half of the blocks (blocks 10–18) in the two common gardens. We analyzed a subset of the samples due to project time constraints. We analyzed foliar chemistry in July because insect diversity and density are highest during July and this month represents the time period during which milkweed chemistry is most likely responsive to plant–herbivore interactions (Agrawal, 2004a) (Appendix A, Table A1).

We analyzed foliar cardenolide concentrations using established methods (Decker et al., 2018; Zehnder & Hunter, 2007). We cut 6 leaf disks with a hole puncher from the fifth leaf pair of each plant and placed the disks in 1 ml of methanol. Samples were stored at −10°C for later cardenolide analysis. We took 6 additional disks from the same leaves to estimate the dry mass of the cardenolide samples. To extract cardenolides, we finely ground the leaf disks in methanol, sonicated the mixture for 1 hr at 60°C, and centrifuged for 6 min. We transferred the supernatant to new 1‐ml Eppendorf tubes and evaporated the samples under vacuum at 45°C until dry. We resuspended the sample in 300 ml of methanol and used reverse‐phase ultra‐performance liquid chromatography (UPLC) on a Waters Acquity UPLC with an Acquity BEH C18 column (1.7 μm, 2.1 × 50 mm, Waters Inc., Milford, MA, USA). We separated and quantified cardenolides with a 0.15 mg/ml digitoxin internal standard (Sigma Chemical Company, St. Louis, Missouri, USA). Each 2 μl injection sample was eluted for 9 min at a constant flow rate of 0.7 ml per minute under a mobile phase of 20% acetonitrile (ACN): 80% water for 3 min followed by a gradient increasing to 45% ACN: 55% water over the remainder of the run. Cardenolides were quantified using a diode array detector scanning between 200 and 300 nm, and we identified cardenolides as peaks with symmetrical absorbance between 216 and 222 nm. To calculate cardenolide concentrations, we took the sums of all separated peak areas, corrected by the concentration of the internal digitoxin standard, and estimated by the dry sample mass.

We measured milkweed latex exudation by collecting latex from the 6 holes cut for the cardenolide samples on preweighed paper disks (Vannette & Hunter, 2011), ensuring no latex was lost and excluding the leaf midrib from the hole punches. Disks were dried in a drying oven at 45°C for 24 hr and then weighed. We measured latex exudation in all 18 blocks in both common gardens.

To analyze foliar carbon and nitrogen concentrations, we collected 2–3 leaves from each plant. Leaves were dried in a drying oven at 45°C and finely ground. Leaf powder was dried again for 24 hr before 2 µg of each sample was transferred to a tin capsule. Carbon and nitrogen concentrations were measured on a ThermoScientific EA 1112 elemental analyzer. We used 99.7% caffeine powder as an external standard.

## 4 STATISTICAL METHODS

Statistical analyses were performed using SAS version 9.4 unless noted otherwise. Because of the complexity of our data (repeated observations on individual milkweeds over the growing season, measured at three locations including two common gardens and maternal genets), we analyzed our data in several ways. First, to examine trait variation among maternal lines over time (repeated observations on individuals), we analyzed each location separately. Second, to explore more explicitly any genotype‐by‐environment interactions, we built models that included data from both common gardens (= environments) simultaneously, but that either averaged or summed trait values over time for each individual milkweed (to remove the repeated measures). Models that included repeated measures and multiple locations simultaneously would not converge.

### 4.1 Separate models for each location

#### 4.1.1 Herbivore resistance and plant growth

We used generalized linear mixed models (PROC GLIMMIX) to assess genetic variation in plant growth and resistance to chewing herbivores (defoliation level). For the common garden analyses, we included block and individual plant ID (nested within maternal line and block) as random variables to account for repeated measures from individual plants and any effects of autocorrelation within blocks. Week was a continuous variable while plant ID, maternal line, and block were class variables. Because plants began to senesce and reduce in size by the end of the season, our models included a quadratic term for time. Overall, our models assessed the effects of maternal line on variation in plant growth (height, leaf number, average base stem diameter) and resistance (defoliation) over the growing season. Significant interactive effects of maternal line and week on character traits represent genetic variation in rates of growth or resistance to herbivory.

Because we collected data from maternal genets only once each month, we used month as a class variable in analyses of variation in growth and resistance of maternal genets. Otherwise, we followed the same model structure as above without a block term.

To analyze data from the reciprocal transplant experiment, we used a similar glimmix model structure but removed the random block term and assessed the effects of maternal line, week, transplant location, and their interactions on plant traits.

#### 4.1.2 Insect populations

Most insect species were encountered too rarely to analyze separately, and aphids were by far the most abundant herbivores that we encountered (Appendix A, Table A1). Accordingly, we restrict our analyses of insect abundance to aphids. However, many individual milkweed plants were never colonized by aphids. Therefore, we first analyzed variation in aphid presence/absence among milkweed maternal lines or genets by performing a generalized linear mixed model (PROC GLIMMIX) using a binomial distribution with a logit link function. This model worked well for one aphid species, *Aphis asclepiadis,* but would not converge for the second species, *Myzocallis asclepiadis*. Therefore, we used a generalized linear model (PROC GENMOD) with a binomial distribution and logit link function for *M. asclepiadis*. Because proc genmod in SAS does not recognize random effects, we designated plant ID (nested within maternal line and block) as a repeated effect and accounted for variation among blocks by assigning block as a main effect.

Next, for those common garden plants that hosted aphids, we used a mixed model (PROC MIXED) and log‐transformed aphid population counts to assess genetic variation for resistance to aphids among maternal lines over time. We held block and plant ID (nested within block and maternal line) as random variables and used plant ID (nested within block and maternal line) as the repeated subject term.

Finally, for the naturally growing maternal genets, we recorded *A. asclepiadis* on only six maternal genets, and only two of those genets hosted *A. asclepiadis* on three or more replicate ramets. Therefore, we did not have enough representation to perform meaningful statistical tests for *A. asclepiadis* population growth among maternal genets. *Myzocallis asclepiaidis* appeared only in the month of August and was observed on 23 maternal ramets. Five of the nine maternal genets represented had three or more ramets with aphids, and therefore, we restricted our analysis to those 5 maternal genets (17 ramets). We log‐transformed *M. asclepiadis* numbers and examined differences in *M. asclepiadis* populations among maternal genets using a general linear model (PROC glm). Because *M. asclepiadis* only appeared in August, no month term or repeated measure was required.

#### 4.1.3 Plant chemistry

To assess genetic variation in foliar chemistry traits (cardenolides, latex, nitrogen, and carbon), we used a generalized linear mixed model (PROC glimmix) and held plant ID, maternal line, and block (for common gardens) as class variables. Block and plant ID (nested with maternal line and block) were random variables. We log‐transformed cardenolide data prior to analysis to meet assumptions of homogeneity of variance. For the maternal genets and reciprocal transplant experiment, we used general linear models (PROC glm). We held plant ID and maternal line as class variables for the maternal genets, and plant ID, maternal line, and location as class variables for the reciprocal transplant milkweeds. Because we only used chemistry data from one date (mid‐July), no week term was required.

#### 4.1.4 Associating genetic variation estimated in common gardens with trait variation in the field

As noted in our predictions (above), we would expect positive correlations in the trait values measured from maternal lines/genets among experimental locations if genetic variation alone dominates trait expression. We therefore correlated milkweed traits (resistance, chemistry, growth) (a) between common gardens, and (b) between each common garden and the natural field population of maternal genets. We first calculated average trait values for each maternal line at each location. We then calculated the slopes of the regressions for each trait across locations, using the means (14 genets/maternal lines) as data points. Regression statistics were calculated using Excel for Mac version 16.33. We calculated average defoliation values for August for each maternal line/genet. We used the data from August because the majority of defoliation occurred in August and defoliation in June and July was rare. For foliar cardenolide concentrations and latex, we used the chemistry data collected in July. To calculate plant growth means, we used data from early and mid‐season, excluding end‐of‐season data due to plant senescence (Appendix B). We calculated initial growth rates of our milkweeds between weeks one and six for the common gardens and between mid‐June and mid‐July for the maternal genets (week 6 of the common garden experiments was the same week as the mid‐July sample of the maternal genets). We calculated initial growth rate for each individual plant (separately for height, leaf number, and diameter) using the following formula: (Week 6 data − Week 1 data)/(Week 1 data) = Initial Growth Rate. We averaged the initial growth rates for each maternal line/genet and compared as described above.

### 4.2 Combining common gardens to explore genotype‐by‐environment interactions

Having calculated seasonal averages for defoliation, foliar chemistry, and initial plant growth (above), we could then build models that included data from both common gardens simultaneously to explore genotype‐by‐environment interactions. For each trait value in turn, we used a general linear mixed model (PROC mixed) with maternal line and location as fixed effects and block as a random effect. We assessed the effect of maternal line (genotype), growing location (environment), and the interaction between maternal line and growing location on trait expression to estimate the strength of genotype‐by‐environment interactions.

While we originally considered maternal line as a fixed effect and block as a random effect in our models because M.D. Hunter has researched the same maternal lines for the past 12 years, we can gain insight into the importance of environmental variation by reversing that designation (Colom & Baucom, 2020). Consequently, we also built general linear mixed models in which we analyzed location and block (nested within location) as fixed effects and held milkweed maternal line as a random effect. Note that we do not include a block‐by‐location interaction in these models as block is nested within location. We analyzed variation in the same trait values as before.

Finally, we explored potential genetic trade‐offs between milkweed growth and resistance to herbivores (Strauss & Agrawal, 1999; Züst et al., 2015). We used principal components analysis to generate a single PCA axis for growth (separately for each common garden & the maternal genets). That is, we combined the initial growth rates of height, leaf number, and stem diameter into a single PCA axis for each common garden/maternal population. We then assessed correlations among milkweed resistance and growth traits (within maternal lines of each common garden and within the maternal genets). We calculated pairwise correlation coefficients among the growth PCA axis, and foliar cardenolide concentrations, latex exudation, foliar C:N ratios, and defoliation.

## 5 RESULTS

### 5.1 Drivers of variation in defoliation

**FIGURE 1:**
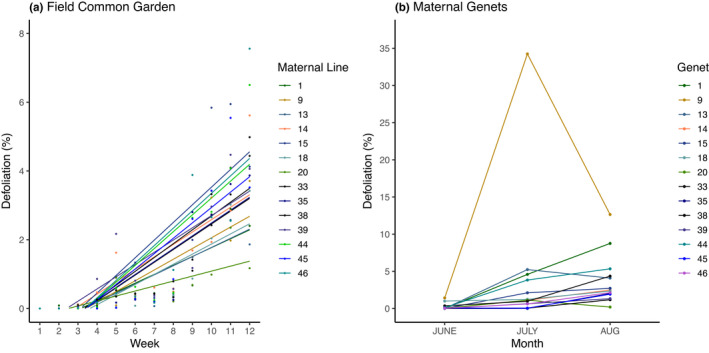
Percent defoliation of milkweeds in (a) a field common garden and (b) their unmanipulated maternal genets. Field common garden maternal lines expressed genetic variation in rate of resistance to herbivory (Week * Maternal line, *F*_13, 2,746_ = 2.30, *p* = .0051). Maternal genets displayed variation in herbivory resistance (Maternal genet * Month, *F*_26, 110_ = 6.56, *p* < .0001). Points represent the mean defoliation for the maternal line/genet for the week/month. Points represent mean defoliation of (a) 18 milkweeds per maternal line, and (b) 5 milkweeds per maternal genet. Lines in (a) are regressions, while lines in (b) are for visual reference only. High July defoliation in maternal genet 9 was due to extensive deer browsing. Note difference in *y*‐axis scale

Maternal lines grown in the field common garden expressed genetic variation for resistance, accumulating defoliation at different rates (Week * Maternal line, *F*_13, 2,746_ = 2.30, *p* = .0051, Figure 1a). Similarly, the maternal genets also varied in resistance, accumulating defoliation at different rates (Maternal genet * Month, *F*_26, 110_ = 6.56, *p* < .0001, Figure 1b). However, defoliation of the maternal lines in the field common garden was uncorrelated with defoliation experienced by their naturally growing maternal genets (*y* = −0.146*x* + 4.2767, *R* ^2^ = 0.0056, *p* = .7992), suggesting that there may be determinants of resistance in addition to genetic variation for naturally growing milkweeds. In support of this, milkweeds in the reciprocal transplant experiment accumulated defoliation at different rates among transplant locations (Week * Location *F*_2, 392_ = 22.53, *p* < .0001) whereas maternal lines were defoliated about equally (*F*_2, 36_ = 0.27, *p* = .7668), irrespective of location (Maternal line * Location, *F*_4, 36_ = 0.20, *p* = .9381). Overall, these results indicate that both maternal line (common garden experiment) and local growing environment (reciprocal transplant experiment) contribute to the variation in resistance to chewing herbivores. As expected, insect densities and defoliation levels were negligible in the greenhouse common garden and are not reported here.

### 5.2 Drivers of variation in insect populations on milkweed

We observed no evidence for genetic variation in *Aphis asclepiadis* colonization among milkweed maternal lines (Maternal line, *F*_13, 221_ = 0.76, *p* = .6976; Week * Maternal line, *F*_13, 2,747_ = 0.60, *p* = .8564) in the field common garden. Likewise, after colonization, we observed no genetic variation in *A. asclepiadis* population densities among maternal lines (Maternal line, *F*_13, 111_ = 1.08, *p* = .3859; Week * Maternal line, *F*_13, 130_ = 1.23, *p* = .2683), indicating that genetic variation may not account for resistance against *A. asclepiadis* population growth. Population sizes of *A. asclepiadis* were too low on both the maternal genets and the reciprocal transplant milkweeds to provide insight.

As with *A. asclepiadis*, we found no evidence for genetic variation in *Myzocallis asclepiadis* colonization among milkweed maternal lines in the field common garden (Maternal line, $\chi_{13}^{2}$ = 12.61, *p* = .4783; Week * Maternal line, $\chi_{13}^{2}$ = 15.48, *p* = .2782). After colonization, we observed no genetic variation in *M. asclepiadis* population levels among maternal lines (Maternal line, *F*_13, 219_ = 1.22, *p* = .2645; Week * Maternal line, *F*_13, 910_ = 1.49, *p* = .1133). In contrast, we did observe variation among five maternal genets (those with aphids present on multiple ramets) in the population densities of *M. asclepiadis* (*F*_4, 12_ = 5.50, *p* = .0095), suggesting that local environment may be a significant determinant in *M. asclepiadis* population growth. Unfortunately, population densities of *M. asclepiadis* were too low on reciprocal transplant milkweeds to provide any additional insight.

### 5.3 Drivers of variation in plant foliar quality

**FIGURE 2:**
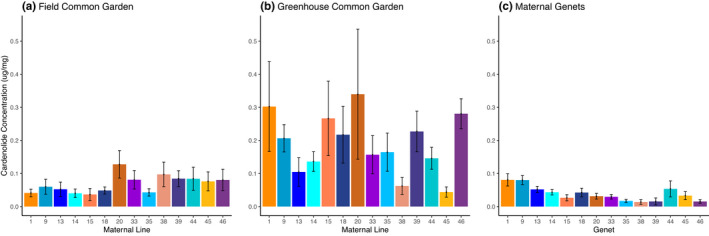
Foliar cardenolide concentrations of milkweed maternal lines in a (a) field common garden, (b) greenhouse common garden, and (c) their maternal genets. Neither the field common garden nor the greenhouse common garden milkweeds displayed genetic variation in cardenolide concentration (*F*_13, 101_ = 1.11, *p* = .3568; *F*_13, 98_ = 1.53, *p* = .1212, respectively). The maternal genets varied in cardenolide concentration (*F*_13, 56_ = 3.42, *p* = .0007). Data were log‐transformed prior to analysis. Bars represent (a, b) 9 milkweeds per maternal line and (c) 5 milkweeds per maternal genet. Error bars are ±1 *SE*

Maternal lines in the field common garden and the greenhouse common garden expressed no genetic variation in foliar cardenolide concentration (*F*_13, 101_ = 1.11, *p* = .3568; *F*_13, 98_ = 1.53, *p* = .1212, respectively; Figure 2a,b). Accordingly, cardenolide concentrations in the field common garden were uncorrelated with those of the greenhouse common garden (*y* = 0.216*x* + 0.1749, *R* ^2^ = 0.004, *p* = .8269). In contrast, foliar cardenolide concentrations did vary among naturally growing maternal genets (*F*_13, 56_ = 3.42, *p* = .0007, Figure 2c). However, the foliar cardenolide concentrations of the maternal genets were uncorrelated with those of either the field or the greenhouse common gardens (*y* = −0.297*x* + 0.0584, *R* ^2^ = 0.1265, *p* = .2120; *y* = 0.0322*x* + 0.0321, *R* ^2^ = 0.0167, *p* = .6592, respectively). Consistent with results from both common gardens, milkweeds in the reciprocal transplant experiment did not vary in cardenolides among maternal lines (Maternal line, *F*_2, 14_ = 1.20, *p* = .3316). However, unlike the maternal genets, cardenolide concentrations did not vary among transplant locations either (Location, *F*_2, 14_ = 0.44, *p* = .6552; Maternal line * Location, *F*_3, 14_ = 0.18, *p* = .9096). In the models in which we combined data from the two common gardens (Table 1a), we again found no evidence of genetic variation in cardenolide concentrations (*F*_13, 199_ = 1.50, *p* = .1185), nor any evidence of a genotype‐by‐environment interaction (*F*_13, 199_ = 1.51, *p* = .1176). However, foliar cardenolide concentrations were much higher in the greenhouse than the field common garden (*F*_1, 16_ = 18.00, *p* = .0006; Figure 2a,b). Moreover, in models in which block was treated as a fixed effect (Table 1b), the importance of the environment in affecting cardenolide concentrations was apparent at two scales: between the two common gardens and among blocks within those gardens (Table 1b).

**TABLE 1:** Results of mixed‐effects models estimating the contributions of genotype, environment, and their interaction on variation in resistance and growth traits of *Asclepias syriaca* in two common gardens

Milkweed trait	Effect	Numerator degrees of freedom	Denominator degrees of freedom	*F*	Pr > *F*
(a)					
Cardenolides	Maternal line	13	199	1.5	0.1185
	Location	1	16	18	**0.0006**
	Location * maternal line	13	199	1.51	0.1176
Latex	Maternal line	13	442	4	**<0.0001**
	Location	1	34	5.12	**0.0302**
	Location * maternal line	13	442	2.02	**0.0177**
Nitrogen	Maternal line	13	208	1.94	**0.0273**
	Location	1	16	4.94	**0.0409**
	Location * maternal line	13	208	1.11	0.3478
Carbon	Maternal line	13	208	4.8	**<0.0001**
	Location	1	16	9.74	**0.0066**
	Location * maternal line	13	208	1.63	0.0786
Height growth	Maternal line	13	442	2.89	**0.0005**
	Location	1	34	166.99	**<0.0001**
	Location * maternal line	13	442	2.84	**0.0006**
Diameter growth	Maternal line	13	442	2.3	**0.0059**
	Location	1	34	22.56	**<0.0001**
	Location * maternal line	13	442	2.87	**0.0005**
Leaf growth	Maternal line	13	442	10.97	**<0.0001**
	Location	1	34	152.6	**<0.0001**
	Location * maternal line	13	442	1.81	**0.0395**
(b)					
Cardenolides	Location	1	212	38.73	**<0.0001**
	Block (location)	16	212	2.21	**0.0059**
Latex	Location	1	455	4.94	**0.0267**
	Block (location)	34	455	0.89	0.6545
Nitrogen	Location	1	221	33.59	**<0.0001**
	Block (location)	16	221	6.79	**<0.0001**
Carbon	Location	1	221	9.34	**0.0024**
	Block (location)	16	221	0.88	0.5888
Height growth	Location	1	455	163.37	**<0.0001**
	Block (location)	34	455	0.98	0.5057
Diameter growth	Location	1	455	21.35	**<0.0001**
	Block (location)	34	455	0.9	0.6348
Leaf growth	Location	1	455	158.15	**<0.0001**
	Block (location)	34	455	1.04	0.4149

Model	Milkweed trait	Fixed effects	Block (location) covariance	Residual
(c)				
A	Cardenolides	Maternal line, location, maternal line * location	0.000238	0.002552
A	Latex	Maternal line, location, maternal line * location	0	1.9334
A	Nitrogen	Maternal line, location, maternal line * location	0.1511	0.3625
A	Carbon	Maternal line, location, maternal line * location	0	1.0573
A	Height growth	Maternal line, location, maternal line * location	0.000578	0.2725
A	Diameter growth	Maternal line, location, maternal line * location	0	0.03199
A	Leaf growth	Maternal line, location, maternal line * location	0.002824	0.6551

Model	Milkweed trait	Fixed effects	Genotype covariance	Residual
(d)				
B	Cardenolides	Block (location)	0.000062	0.002626
B	Latex	Block (location)	0.1593	2.0018
B	Nitrogen	Block (location)	0.01881	0.3649
B	Carbon	Block (location)	0.2209	1.1026
B	Height growth	Block (location)	0.01394	0.2868
B	Diameter growth	Block (location)	0.001108	0.03381
B	Leaf growth	Block (location)	0.181	0.6703

Note

In (a), maternal line, garden location, and their interaction are fixed effects, and block (nested within location) is a random effect. In (b), maternal line is a random effect, while garden location and block (nested within location) are fixed effects. (C) AND (D) show the covariance parameters for models represented in (A) and (B). “Location” represents a common garden grown in a field or a greenhouse. Latex exudation and foliar concentrations of nitrogen, carbon, and cardenolide were measured in July. Initial growth rates were used for milkweed height, stem diameter, and leaves (see text for details). Significant results are in boldface.

**FIGURE 3:**
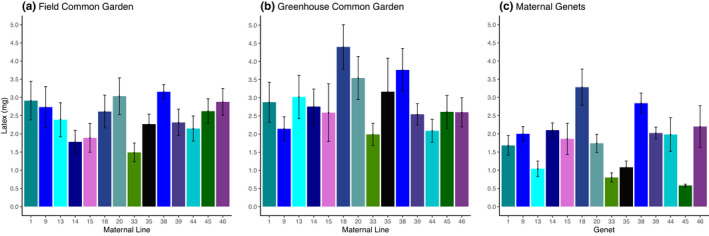
Mean foliar latex exudation of maternal lines in a (a) field common garden, (b) greenhouse common garden, and (c) their maternal genets. Maternal lines displayed genetic variation in foliar latex exudation in the field and greenhouse common gardens (*F*_13, 221_ = 3.89, *p* < .0001; *F*_13, 221_ = 2.49, *p* = .0034, respectively), and foliar latex exudation varied among the maternal genets (*F*_13, 56_ = 5.64, *p* < .0001). Bars represent (a, b) 18 milkweeds per maternal line and (c) 5 milkweeds per maternal genet. Error bars are ±1 *SE*

We observed genetic variation in foliar latex exudation in both the field and greenhouse common gardens (*F*_13, 221_ = 3.89, *p* < .0001; *F*_13, 221_ = 2.49, *p* = .0034, respectively; Figure 3a,b). However, latex exudation was uncorrelated among maternal lines between the two common gardens (*y* = 0.3414*x* + 0.0019, *R* ^2^ = 0.108, *p* = .2512). That lack of correlation likely reflects a significant interaction between the environment (greenhouse versus field) and maternal line that we detected in the model containing both common gardens (Maternal line by location interaction: *F*_13, 442_ = 5.12, *p* = .0177, Table 1a). Maternal genets also varied in foliar latex exudation (*F*_13, 56_ = 5.64, *p* < .0001, Figure 3c), but foliar latex in the maternal genets was uncorrelated with either the field or greenhouse common gardens (*y* = 0.1645*x* + 0.0014, *R* ^2^ = 0.0151, *p* = .6756; *y* = 0.4814*x* + 0.0005, *R* ^2^ = 0.1396, *p* = .1882, respectively). Unlike the effects on foliar cardenolides, we observed no small‐scale effect of block on latex exudation (Table 1b).

**FIGURE 4:**
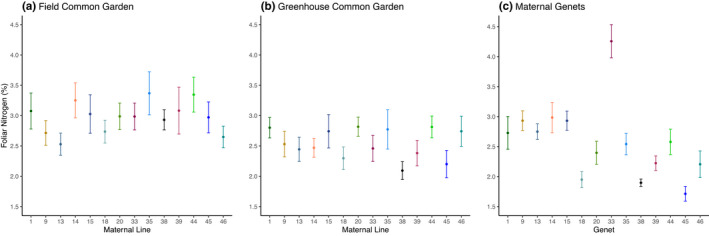
Mean percent foliar nitrogen concentration of maternal lines in a (a) field common garden, (b) greenhouse common garden, and (c) their maternal genets. Neither the field nor greenhouse common garden milkweeds displayed genetic variation in percent nitrogen (*F*_13, 104_ = 1.38, *p* = .1821; *F*_13, 104_ = 1.73, *p* = .0659, respectively). The maternal genets varied in percent nitrogen (*F*_13, 56_ = 11.21, *p* < .0001). Points are means of (a, b) 9 milkweeds per maternal line and (c) 5 milkweeds per maternal genet. Error bars are ±1 *SE*

We did not observe genetic variation in foliar nitrogen concentration in the field or greenhouse common gardens (*F*_13, 104_ = 1.38, *p* = .1821; *F*_13, 104_ = 1.73, *p* = .0659, respectively; Figure 4a,b), and foliar nitrogen concentrations between the two common gardens were uncorrelated (*y* = 0.262*x* + 1.759, *R* ^2^ = 0.0732, *p* = .3497). However, foliar nitrogen concentrations varied substantially among the maternal genets (*F*_13, 56_ = 11.21, *p* < .0001, Figure 4c), suggesting that local environment is an important driver of foliar nitrogen concentrations. Accordingly, foliar nitrogen concentration in the maternal genets was not predicted by those of the maternal lines in either of the common gardens (Field, *y* = 0.2276*x* + 1.899, *R* ^2^ = 0.0082, *p* = .7575; Greenhouse, *y* = 0.7734*x* + 0.6131, *R* ^2^ = 0.0893, *p* = .2993). Nonetheless, in models in which we included both common gardens simultaneously (Table 1a), we observed significant, albeit weak, effects of both maternal line (*F*_13, 208_ = 1.94, *p* = .0273) and garden location (*F*_1, 16_ = 4.94, *p* = .0409) on variation in foliar nitrogen concentration. There was no significant genotype‐by‐environment interaction (Table 1a). However, in models in which block was treated as a fixed effect (Table 1b), the importance of the environment in affecting foliar nitrogen concentrations was apparent at two scales: between the two common gardens and among blocks within those gardens (Table 1b).

**FIGURE 5:**
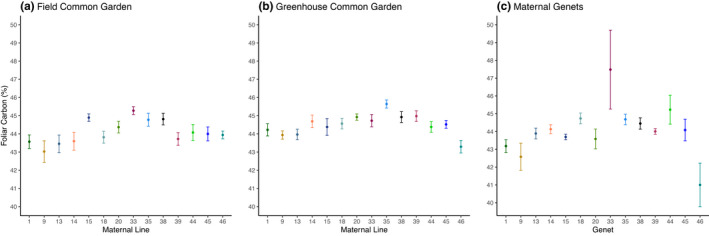
Mean percent foliar carbon concentration of maternal lines in a (a) field common garden, (b) greenhouse common garden, and (c) their maternal genets. The milkweed in the field and greenhouse common gardens displayed genetic variation in percent carbon (*F*_13, 104_ = 3.06, *p* = .0007; *F*_13, 104_ = 3.46, *p* = .0002). The maternal genets also varied in percent foliar carbon (*F*_13, 56_ = 3.23, *p* = .0011). Points are means of (a, b) 9 milkweeds per maternal line and (c) 5 milkweeds per maternal genet. Error bars are ±1 *SE*

In contrast to foliar nitrogen concentrations, maternal lines expressed substantial genetic variation in foliar carbon concentration in both the field and greenhouse common gardens (*F*_13, 104_ = 3.06, *p* = .0007; *F*_13, 104_ = 3.46, *p* = .0002, respectively; Figure 5a,b). However, the foliar carbon concentrations of the maternal lines were uncorrelated between the two common gardens (*y* = 0.4262*x* + 25.713, *R* ^2^ = 0.2491, *p* = .0693, Appendix A, Table A2), suggesting a role for environmental variation in determining foliar carbon concentrations, and a potential genotype‐by‐environment interaction. The important roles of both genotype and environment were confirmed in the model that contained both common gardens (Table 1a), in which maternal line (*F*_13, 208_ = 4.80, *p* < .0001) and garden location (*F*_1, 16_ = 9.74, *p* = .0066) were both significant predictors of foliar carbon concentration. Their interaction was marginally nonsignificant (*F*_13, 208_ = 1.63, *p* = .0786, Table 1a). The maternal genets also varied in foliar carbon concentrations (*F*_13, 56_ = 3.23, *p* = .0011, Figure 5c), and foliar carbon concentrations in the maternal genets were correlated with both the field and the greenhouse common gardens (*y* = 1.195*x* − 8.6549, *R* ^2^ = 0.3032, *p* = .0413; *y* = 1.5071*x* − 23.026, *R* ^2^ = 0.3517, *p* = .0254, respectively), suggesting that genetic variation is a major driver of differences in foliar carbon concentrations among field‐grown milkweeds.

### 5.4 Plant growth

The primary goal of our research was to study the origins of phenotypic variation in resistance and resistance traits in milkweed. However, during our experiments, we also estimated plant size (height, leaf number, diameter) whenever we counted insects. We then analyzed our size estimates using the same techniques described above. Because measuring phenotypic variation in plant size was not a primary goal of our study, we restrict the presentation of these analyses to Table 1, with a more detailed description of results in Appendix B. We also assessed correlations among milkweed growth and resistance traits within each common garden and within the maternal genets. A table of all correlation results may be found in Appendix A, Table A2.

## 6 DISCUSSION

Our results suggest that milkweed resistance traits are influenced substantially by their growing environment and by genotype‐by‐environment interactions and that standing genetic variation plays a smaller role in phenotypic variation in this population of common milkweed. We recorded considerable phenotypic variation in traits of the maternal genets in the naturally growing population, but only a subset of those traits varied among maternal lines in the common gardens. We observed significant effects of growing environment and genotype‐by‐environment interactions on trait expression, with evidence of G × E interactions in all recorded herbivore resistance traits except for foliar nitrogen, foliar carbon, and foliar cardenolide concentrations (Table 1a). Our results indicate that growing location, either alone or while interacting with genotype, is responsible for generating much of the trait variation within our single milkweed population.

Similar to previous studies of milkweed (Agrawal, 2004b) and other plant species (Fritz & Price, 1988; Maddox & Root, 1987), we observed evidence of genetic variation in herbivore resistance (Figure 1). However, defoliation of the maternal genets and that of the maternal lines did not correlate, suggesting that the growing environment may determine the relative resistance to herbivory expressed by milkweed genets (G × E). While identifying G × E interactions within our study population is unsurprising (Barrett & Agrawal, 2004; Johnson & Agrawal, 2005; Maddox & Root, 1987), our work contrasts with previous studies which suggest that genetic variation plays a relatively larger role in phenotypic variation at smaller scales (Johnson & Agrawal, 2005). We both sourced our seeds and sampled genetic and phenotypic variation within a single population in a small area, yet environmental influences on phenotypic variation were generally stronger than those of genotype. As others have noted, understanding how spatial scale influences the expression of genotype is a major goal of evolutionary ecology (Tack et al., 2012).

At local scales, if certain maternal genets have experienced more herbivory over time than others, the effects of transgenerational resistance to herbivory (Agrawal, 2002; Holeski et al., 2012) could contribute to the lack of correlation between our maternal genets and their maternal lines. More generally, non‐Mendelian parental effects can exert a strong influence on plant offspring phenotype (Roach & Wulff, 1987; Weiner et al., 2009), whereby the environment experienced by maternal plants is reflected in offspring phenotype. Multiple generations of common garden plants would be required to separate unequivocally non‐Mendelian parental effects from genetic effects in our study (Mousseau & Fox, 1998). However, in our design, strong environmentally based parental effects from field‐grown maternal genets would tend to inflate the importance of maternal line as a main effect on phenotypic variation in their offspring. In contrast, current growing environment (the type of common garden, and the block within those gardens) generally explained more phenotypic variation than did maternal line alone, suggesting that environmental factors may be stronger than any maternal effects (De Long et al., 2019). Beyond maternal effects, ontogeny is an important determinant of plant resistance traits (Barrett & Agrawal, 2004; Muola et al., 2010), and the difference in ontogenetic stage between our established genets and their first‐year maternal lines very likely influenced resistance traits in our study (Yang et al., 2020). Because milkweed maternal genets in our study are at least 12 years old (M.D. Hunter, *personal communication*), differences in age of the maternal lines and the maternal genets may affect their relative resistance to defoliation as well as other aspects of phenotype.

Cardenolide concentrations were influenced mainly by the growing environment of the milkweed (Table 1). This result aligns with a robust body of literature that describes how environmental variation influences foliar cardenolide concentrations (Decker et al., 2018; Faldyn et al., 2018; Matiella, 2012; Vannette & Hunter, 2011). However, we did not detect genetic variation or G x E interactions in cardenolide concentrations, unlike other studies (Agrawal, 2004a; Agrawal & Hastings, 2019b; Vannette & Hunter, 2011). Previous work has shown that the population of *A. syriaca* at UMBS differs in resistance traits from other milkweed populations at regional scales (Andrews, 2015), indicating that, while our single study population may lack substantial genetic variation, we might expect more genetic variation among regional populations. We also observed highly positive correlations between defoliation and cardenolide concentration in the naturally growing maternal genets (Appendix A, Table A2), supporting the body of evidence showing cardenolide induction by herbivory (Malcolm & Zalucki, 1996; Rasmann et al., 2009, but see Zehnder & Hunter, 2007). However, other studies have found that plant damage and cardenolides do not correlate (Agrawal, 2005) and that cardenolides concentrations are determined by G × E interactions (Vannette & Hunter, 2011). These contrasting results illustrate the complexity of plant defense phenotypes and their underlying causes. Certain maternal genets may be expressing higher levels of cardenolides due to priming of chemical defense against herbivory over multiple years (Frost et al., 2008). Thus, the data that we collected from field plants likely includes responses to drivers of foliar chemistry that we neither measured nor controlled during our study.

Foliar cardenolide concentrations from maternal lines were also three‐ to fourfold times higher in the greenhouse than in the field common garden, (Figure 2a,b), amply demonstrating how growing environment can influence the expression and phenotypic plasticity of milkweed resistance traits. The increase in toxicity in the greenhouse milkweeds is likely due to the elevated temperatures in the greenhouse (Faldyn et al., 2018). Interestingly, the phenotypic plasticity in milkweed toxicity that we recorded could provide a potential increase in herbivore resistance in an increasingly warmer environment.

Although we observed genetic variation in latex exudation in both common gardens, latex exudation was also affected by growing environment and G × E interactions (Figure 3, Table 1). Similarly, other studies have also identified G × E interactions in milkweed latex exudation (Agrawal & Hastings, 2019a). Latex exudation of maternal lines in the field common garden was negatively genetically correlated with defoliation (Appendix A, Table A2), supporting much evidence that latex is an effective defense against chewing herbivores (Agrawal & Van Zandt, 2003; Van Zandt & Agrawal, 2004). Curiously, defoliation and latex exudation were uncorrelated in the maternal genets (Appendix A, Table A2). However, other herbivores may be at play: latex production that is induced by monarch caterpillar herbivory can be returned to premonarch levels by simultaneous root herbivory (Rasmann et al., 2009), reminding us that unrecorded belowground herbivory may confound correlations among resistance traits and herbivore activity (Hunter, 2001).

Foliar carbon concentration was the only recorded milkweed trait that correlated between the maternal lines and the maternal genets, suggesting a strong genetic component to its expression (Figure 5, Table 1). However, the lack of correlation in foliar carbon between the field and greenhouse maternal lines again demonstrates the phenotypic plasticity of common milkweed. In contrast to foliar carbon, foliar nitrogen concentrations were strongly influenced by the growing environment (Figure 4, Table 1). Indeed, interactions with environmental conditions have been shown previously to generate variability in foliar nitrogen concentrations. For example, elevated temperatures can increase foliar nitrogen in milkweed (Couture et al., 2015), and both above‐ and belowground herbivore attack cause milkweed to preferentially allocate nitrogen away from sites of damage and into stems (Tao & Hunter, 2013), altering the C:N ratio of the plant tissue. While we observed no correlation between foliar C:N ratios and aboveground defoliation (Appendix A, Table A2), unrecorded belowground herbivory could be a driver of the observed variation.

We found a lack of genetic variation for resistance to aphid colony establishment or growth for either aphid species. Nonetheless, the naturally growing maternal genets displayed variation in resistance to *M. asclepiadis* colony growth, suggesting that the local environment has a strong role in shaping the dynamics of *M. asclepiadis*. However, a previous study found that genetic variation *within* common milkweed populations influences variation in *A. asclepiadis* abundance 5.5‐fold under conditions of interspecific competition among aphids (Smith et al., 2008). Beyond milkweed, *among*‐population genetic variation in *Oenothera biennis*, accounted for 19.62% of arthropod abundance and 11.01% of arthropod species richness (Johnson & Agrawal, 2005). The differences in these results highlight that the level to which genetic variation influences insect population dynamics can be specific to a given population or growing location (Tack et al., 2012), and because we sampled from a small population and grew our common gardens in the same environment, this may explain why we identified a limited role of genetic variation in most traits.

In conclusion, the results from our work indicate that standing genetic variation alone has a limited role in generating phenotypic variation within this small population of common milkweed. Our results highlight the notion that spatial scale and population size should always be addressed explicitly when studying genetic variation. Here, environmental variation and G x E interactions act as the primary drivers of milkweed phenotypic variation, and phenotypic plasticity is prevalent (Table 1). Although we did not assess fitness or adaptation in our study, recent work suggests that phenotypic plasticity may act as a form of “trait re‐adaptation” to ancestral environmental conditions (Ho et al., 2020). Given milkweed diversified in lower latitudes during a hotter era (Agrawal et al., 2009) and milkweeds growing at lower latitudes are often more toxic (Rasmann & Agrawal, 2011), the phenotypic plasticity of milkweed toxicity shown in our results and in other studies (Faldyn et al., 2018) could suggest that this response is a “re‐adaptation” to the hotter temperatures of ancestral growing environments. If “predictability favors plasticity” (Alpert & Simms, 2002), then a change in environment toward the ancestral environment may indeed favor the phenotypic plasticity that we observed. Our results demonstrate that G × E interactions and environmental variation are important drivers of milkweed phenotypic variation, especially at small population scales. Our findings add to a robust body of literature studying the mechanisms behind phenotypic variation in milkweed and complement recent evidence that physical location is a stronger determinant of milkweed phenotype than previously believed (Ricono et al., 2020).

## CONFLICT OF INTEREST

The authors have no sources of conflict of interest.

## AUTHOR CONTRIBUTIONS

**Abigail S. Potts:** Conceptualization (equal); data curation (equal); formal analysis (equal); funding acquisition (supporting); investigation (lead); methodology (equal); project administration (lead); writing‐original draft (lead); writing‐review & editing (equal). **Mark D. Hunter:** Conceptualization (equal); data curation (equal); formal analysis (equal); funding acquisition (lead); investigation (supporting); methodology (equal); project administration (supporting); writing‐original draft (supporting); writing‐review & editing (equal).

## Figures and Tables

**FIGURE B1:**
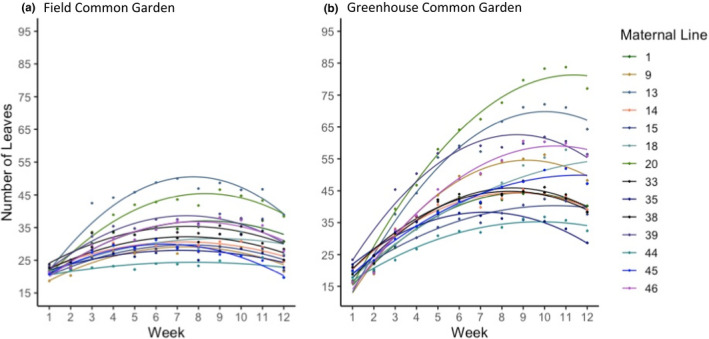
Mean leaf production throughout the season of milkweed maternal lines in (a) a field common garden and (b) a greenhouse common garden. The maternal lines in both the field common garden and greenhouse common displayed genetic variation in rate of leaf production and senescence (Week * Week * Maternal line *F*_14, 2,733_ = 25.18, *p* < .0001; Week * Week * Maternal line *F*_14,2,744_ = 35.66, *p* < .0001, respectively). Points represent the mean height of 18 milkweeds of a maternal line for each week

**FIGURE B2:**
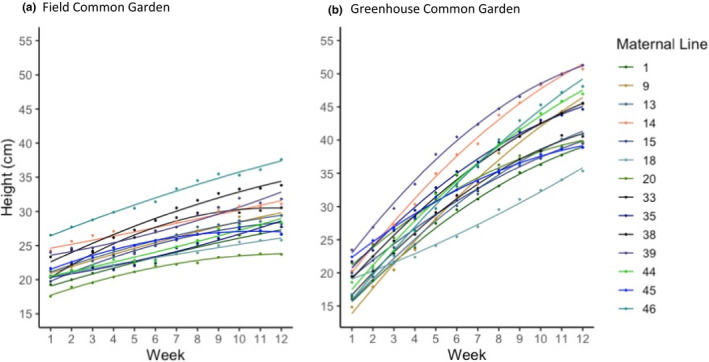
Mean height (cm) of milkweed maternal lines throughout the season in a (a) field common garden and a (b) greenhouse common garden. Maternal lines in the field and greenhouse common gardens displayed genetic variation in height growth rate and senescence rate (Week * Week * Maternal line, *F*_14, 2,733_ = 2.83, *p* = .0003; Week * Week * Maternal line, *F*_14, 2,744_ = 10.99, *p* < .0001, respectively). Points represent the mean height of 18 milkweeds of a maternal line for each week

**FIGURE B3:**
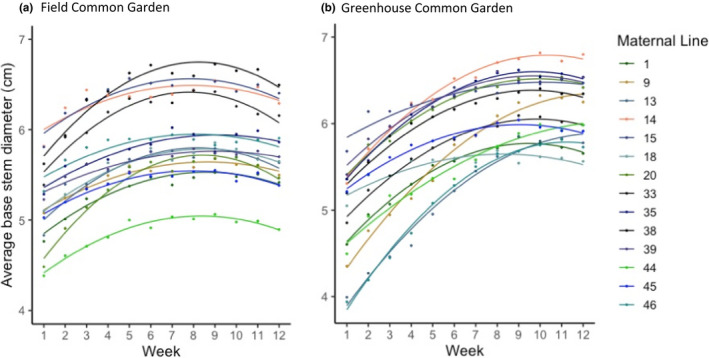
Milkweed base stem diameter (cm) throughout the season in a (a) field common garden and a (b) greenhouse common garden. Maternal lines in the field common garden displayed genetic variation in base stem diameter growth rate and senescence rate (Week * Week * Maternal line, *F*_14, 2,733_ = 23.29, *p* < .0001), as did maternal lines in the greenhouse common garden (Week * Week * Maternal line, *F*_14, 2,744_ = 16.33, *p* < .0001). Points represent the mean height of 18 milkweeds of a maternal line for each week

## Data Availability

Data are made available in the Dryad Digital Repository https://doi.org/10.5061/dryad.rv15dv47x.

## APPENDIX A

**TABLE A1:** Total arthropods counted on milkweed maternal lines in the field common garden in June, July, and August

Group	Arthropod	June	July	August
*Asclepias* specialist	*Danaus plexippus* larvae	15	41	16
*Asclepias* specialist	*Danaus plexippus* eggs	13	84	3
*Asclepias* specialist	*Rhyssomatus lineaticollis*	4	5	7
*Asclepias* specialist	*Tetraopes tetrophthalmus*	0	0	10
*Asclepias* specialist	*Aphis asclepiadis*	541	15,977	1604
*Asclepias* specialist	*Myzocallis asclepiadis*	15	10,170	25,744
*Asclepias* specialist	*Liriomyza asclepiadis*	5	514	522
*Asclepias* specialist	*Lygaeus kalmii*	2	45	6
*Asclepias* specialist	*Euchaetes egle* larvae	0	607	134
Predator	Spiders	99	230	195
Predator	Coccinelidae	1	22	35
Predator	Miridae	0	14	4
Predator	Lacewing larvae	0	4	2
Predator	Syrphid fly larvae	0	6	2
	Total	695	27,719	28,284

Note

Arthropods were identified to level listed above.

**TABLE A2:** Correlations between milkweed resistance and growth traits within each common garden and within the maternal genets

Location	Variable *X*	Variable *Y*	Equation	*R*‐squared	*p*‐value
Field Common Garden	Growth PCA	Cardenolides	*y* = 0.0093*x* + 0.0681	*R* ^2^ = 0.2348	*p* = .0791
Greenhouse Common Garden	Growth PCA	Cardenolides	*y* = 0.0041*x* + 0.1896	*R* ^2^ = 0.0051	*p* = .8084
Maternal Genets	Growth PCA	Cardenolides	*y* = 0.0054*x* + 0.0382	*R* ^2^ = 0.1013	*p* = .2674
Field Common Garden	Growth PCA	Defoliation	*y* = −0.0115*x* + 0.4447	*R* ^2^ = 0.0026	*p* = .8613
Maternal Genets	Growth PCA	Defoliation	*y* = 3.1675*x* + 3.9864	*R* ^2^ = 0.2171	*p* = .0931
Field Common Garden	Growth PCA	Latex	*y* = 9E−05*x* + 0.0024	*R* ^2^ = 0.0576	*p* = .4087
Greenhouse Common Garden	Growth PCA	Latex	*y* = −0.0002*x* + 0.0029	*R* ^2^ = 0.1455	*p* = .1784
Maternal Genets	Growth PCA	Latex	*y* = −0.0002*x* + 0.0018	*R* ^2^ = 0.0846	*p* = .3130
Field Common Garden	Growth PCA	C:N	*y* = 0.434*x* + 14.888	*R* ^2^ = 0.2460	*p* = .0713
Greenhouse Common Garden	Growth PCA	C:N	*y* = −0.3684*x* + 17.683	*R* ^2^ = 0.0971	*p* = .2782
Maternal Genets	Growth PCA	C:N	*y* = −2.1629*x* + 17.927	*R* ^2^ = 0.5045	***p* = .0044**
Field Common Garden	Cardenolides	Defoliation	*y* = −2.6695*x* + 0.6752	*R* ^2^ = 0.1653	*p* = .7168
Maternal Genets	Cardenolides	Defoliation	*y* = 208.58*x* − 5.2871	*R* ^2^ = 0.8878	***p* = .0094**
Field Common Garden	Latex	Defoliation	*y* = −295.3*x* + 1.1569	*R* ^2^ = 0.2826	***p* = .0504**
Maternal Genets	Latex	Defoliation	*y* = 636.13*x* + 2.8414	*R* ^2^ = 0.0029	*p* = .8558
Field Common Garden	C:N	Defoliation	*y* = −0.0274*x* + 0.8756	*R* ^2^ = 0.0100	*p* = .7335
Maternal Genets	C:N	Defoliation	*y* = −0.7372*x* + 17.474	*R* ^2^ = 0.1100	*p* = .2466
Field Common Garden	Cardenolides	Latex	*y* = 0.0072*x* + 0.002	*R* ^2^ = 0.1497	*p* =.1717
Greenhouse Common Garden	Cardenolides	Latex	*y* = 0.0004*x* + 0.0028	*R* ^2^ = 0.0033	*p* = .8451
Maternal Genets	Cardenolides	Latex	*y* = −0.001*x* + 0.0018	*R* ^2^ = 0.0009	*p* = .9200
Field Common Garden	C:N	Latex	*y* = 0.0001*x* + 0.0006	*R* ^2^ = 0.0915	*p* = .2933
Greenhouse Common Garden	C:N	Latex	*y* = 0.0001*x* + 0.0006	*R* ^2^ = 0.1222	*p* = .2205
Maternal Genets	C:N	Latex	*y* = 5E−05*x* + 0.0008	*R* ^2^ = 0.0838	*p* = .3153
Field Common Garden	C:N	Cardenolides	*y* = 0.0011*x* + 0.0523	*R* ^2^ = 0.0024	*p* = .8692
Greenhouse Common Garden	C:N	Cardenolides	*y* = −0.0341*x* + 0.7928	*R* ^2^ = 0.4891	***p* = .0054**
Maternal Genets	C:N	Cardenolides	*y* = −0.0019*x* + 0.0724	*R* ^2^ = 0.1173	*p* = .2307

Note

Significant results are in boldface.

## APPENDIX B

### Plant growth

The maternal lines grown in both the field and greenhouse common garden expressed genetic variation in leaf production and senescence rate (Week * Week * Maternal line, *F*_14, 2,733_ = 25.18, *p* < .0001; Week * Week * Maternal line, *F*_14, 2,744_ = 35.66, *p* < .0001, respectively; Figure B1a,b). When we combined the data from both common gardens (Table 1a), we found evidence of a genotype‐by‐environment interaction for initial leaf growth rates (*F*_13, 442_ = 1.81, *p* = .0395). Initial rates of leaf growth were correlated between the two gardens (*y* = 1.8967*x* + 0.4142, *R* ^2^ = 0.8383, *p* < .0001). Maternal genets also varied in leaf production rates (Maternal line * Month, *F*_26, 110_ = 2.71, *p* = .0001), but maternal genet leaf production was uncorrelated with leaf production in either common garden (Field, *y* = 0.8186*x* + 0.9316, *R* ^2^ = 0.0254, *p* = .5865; Greenhouse, *y* = 0.352*x* + 0.8101, *R* ^2^ = 0.0717, *p* = .3546).

In contrast to the common gardens, maternal lines in the reciprocal transplant experiment had similar rates of leaf production (Week * Maternal line, *F*_2, 392_ = 1.45, *p* = .2358; Maternal line * Location, *F*_4, 36_ = 0.20, *p* = .9381). Notably, leaf production rates varied among transplant locations (Week * Location, *F*_2, 392_ = 22.53, *p* < .0001), indicating the importance of local resources for milkweed growth rates.

Maternal lines in both the field and greenhouse common garden displayed genetic variation in height growth and senescence rate (Week * Week * Maternal line, *F*_14, 2,733_ = 2.83, *p* = .0003; Week * Week * Maternal line, *F*_14, 2,744_ = 10.99, *p* < .0001; Figure B2a,b). However, the initial height growth rates of the common gardens were uncorrelated (*y* = 0.0831*x* + 0.7929, *R* ^2^ = 0.0005, *p* = .9425). Indeed, in the model in which we combined the data from the two common gardens (Table 1a), we again found evidence of a genotype‐by‐environment interaction in milkweed initial height growth rates (*F*_13, 442_ = 2.84, *p* = .0006). Maternal genets varied in height (*F*_13, 56_ = 6.43, *p* < .0001) and height growth rate (Maternal line * Month, *F*_26, 110_ = 2.71, *p* = .0002), but initial height growth rates of the maternal genets were not predicted by the field or the greenhouse common gardens (*y* = 0.8186*x* + 0.9316, *R* ^2^ = 0.0254, *p* = .5865; *y* = 0.352*x* + 0.8101, *R* ^2^ = 0.0717, *p* = .3546, respectively).

Plant heights in the reciprocal transplant experiment did not vary among maternal lines or maternal transplant locations (Week * Maternal line, *F*_2, 395_ = 2.88, *p* = .0576; Location, *F*_2, 36_ = 1.25, *p* = .2983; Maternal line * Location, *F*_4, 36_ = 0.09, *p* = .9858), and height did not vary among transplant locations (Week * Location, *F*_2, 395_ = 1.46, *p* = .2334). It is possible that plants were still too small for differences to emerge by the end of the growing season.

Maternal lines in both the field and greenhouse common gardens expressed genetic variation in base stem diameter growth and senescence rates (Week * Week * Maternal line, *F*_14, 2,733_ = 23.29, *p* < .0001; Week * Week * Maternal line, *F*_14, 2,744_ = 16.33, *p* < .0001, respectively; Figure B3a,b), but were uncorrelated between gardens (*y* = −0.217*x* + 0.2535, *R* ^2^ = 0.0158, *p* = .6689). We also found evidence of a genotype‐by‐environment interaction in stem diameter initial growth rates when we combined the data of the two common gardens (*F*_13, 442_ = 2.87, *p* = .0005; Table 1a), supporting the finding of a lack of correlation between the two gardens. Maternal genets also varied in base stem diameter (*F*_13, 56_ = 4.54, *p* < .0001) but not base stem diameter growth rates (Maternal line * Month, *F*_26, 110_ = 1.34, *p* = .1495). Neither the field common garden nor the greenhouse common garden stem diameter initial growth rates were correlated with those of the maternal genets (*y* = −0.0565*x* + 0.11, *R* ^2^ = 0.002, *p* = .8797; *y* = 0.1464*x* + 0.0693, *R* ^2^ = 0.0399, *p* = .4933, respectively).
